# Detection of medical text semantic similarity based on convolutional neural network

**DOI:** 10.1186/s12911-019-0880-2

**Published:** 2019-08-07

**Authors:** Tao Zheng, Yimei Gao, Fei Wang, Chenhao Fan, Xingzhi Fu, Mei Li, Ya Zhang, Shaodian Zhang, Handong Ma

**Affiliations:** 1 Institute of Image Communication and Networking, Shanghai Jiao Tong University, Shanghai, China; 2 Tongren Hospital, Shanghai Jiao Tong University, Shanghai, China; 3 Synyi Research, Shanghai, China; 4 APEX Data and Knowledge Management Lab, Shanghai Jiao Tong University, Shanghai, China; 5 Department of Healthcare Policy and Research, Weill Cornell Medicine, New York, USA; 6 Shanghai Jiao Tong University, Shanghai, China

**Keywords:** Text similarity, Convolutional neural network, LIME, Natural language processing

## Abstract

**Background:**

Imaging examinations, such as ultrasonography, magnetic resonance imaging and computed tomography scans, play key roles in healthcare settings. To assess and improve the quality of imaging diagnosis, we need to manually find and compare the pre-existing reports of imaging and pathology examinations which contain overlapping exam body sites from electrical medical records (EMRs). The process of retrieving those reports is time-consuming. In this paper, we propose a convolutional neural network (CNN) based method which can better utilize semantic information contained in report texts to accelerate the retrieving process.

**Methods:**

We included 16,354 imaging and pathology report-pairs from 1926 patients who admitted to Shanghai Tongren Hospital and had ultrasonic examinations between 1st May 2017 and 31st July 2017. We adapted the CNN model to calculate the similarities among the report-pairs to identify target report-pairs with overlapping body sites, and compared the performance with other six conventional models, including keyword mapping, latent semantic analysis (LSA), latent Dirichlet allocation (LDA), Doc2Vec, Siamese long short term memory (LSTM) and a model based on named entity recognition (NER). We also utilized graph embedding method to enhance the word representation by capturing the semantic relations information from medical ontologies. Additionally, we used LIME algorithm to identify which features (or words) are decisive for the prediction results and improved the model interpretability.

**Results:**

Experiment results showed that our CNN model gained significant improvement compared to all other conventional models on area under the receiver operating characteristic (AUROC), precision, recall and F1-score in our test dataset. The AUROC of our CNN models gained approximately 3–7% improvement. The AUROC of CNN model with graph-embedding and ontology based medical concept vectors was 0.8% higher than the model with randomly initialized vectors and 1.5% higher than the one with pre-trained word vectors.

**Conclusion:**

Our study demonstrates that CNN model with pre-trained medical concept vectors could accurately identify target report-pairs with overlapping body sites and potentially accelerate the retrieving process for imaging diagnosis quality measurement.

## Background

Imaging examinations are common, as well as efficient, diagnostic tools in clinical practice worldwide. Radiologists or sonographers perform examinations, observe images and write reports for meaningful findings, conclusions and opinions. Imaging examinations are highly operator-dependent modality, and many factors influence the interpretation of the images, such as patients’ demographics, current health status and medical histories. There could be discrepancies in such complicated and heterogeneous information (e.g., the diagnosis in patient’s radiology report is different than the one his/her really has), which may lead to imprecise clinical decisions [1]. Although such discrepancies could be inevitable due to the complexity of imaging-diagnosis, quality measurement and improvement are still needed to minimize avoidable error via a manual verification process. A common objective and standardized verification process is to retrospectively compare the reports of prior imaging and follow-up pathology examinations [2]. However, only few patients receiving imaging examinations on certain body site will have surgical or pathologic biopsy on the same site. To find these patients, quality control staff will regularly and manually review electrical medical records (EMRs) and scan related examination reports, which is inefficient and time consuming. In this study, we propose a machine learning based approach to retrieve these patients from EMRs more efficiently.

Formally, we aim to predict which of the provided report pairs, imaging report and pathologic report, contain overlapping body sites or regions based on their textual semantic similarity. It is slightly different from conventional text similarity cases where researchers care about if two sentences have the same meaning [3, 4]. We care about similar “body sites”, site on the patient’s body where the anomaly has been detected, rather than similar syntax or semantics in general. For example, Table 1 shows a report-pair in our study with original Chinese and translated English. This report-pair contains overlapping body sites — parotid gland, but only the pathology report mentions “parotid gland” (腮腺), and the imaging report describes the condition about “maxillofacial region” (颌面部). Parotid gland is anatomically located in the maxillofacial regions, and thus, the report-pair has similar “body site” and should be picked up. In spite of their different forms, the methodology remains unchanged — the model should extract features from texts, calculate and judge if the pairs have enough common information with certain criteria, and then assign the pairs to certain nominal categories (match or mismatch).

**Table 1:** A report-pair in this study

Language	Imaging report content	Pathologic report content
English	The solid hypoechoic area of the subcutaneous tissues of maxillofacial region is 14.6 mm × 10.4 mm and covered with a capsule. The boundary is clear and the shape is regular.	The specimen for pathological examination contains one mass. The size of mass is 1.2 × 1 × 1 cm, the color is gray red and the capsule is complete. (Parotid gland) favor a diagnosis of pleomorphic adenoma. The lesion contains abundant cells without a clear limit out of the surrounding tissue.
Chinese	颌面部所指处皮下见实质性低回声区14.6 mm × 10.4 mm, 边界清, 有包膜, 形态规则。	肿块一枚, 大小1.2*1*1 cm, 灰红色, 包膜完整。(腮腺)多形性腺瘤, 细胞丰富, 与周围组织分界不清。

We believe that developing a well-designed semantic similarity algorithm should consider three main aspects: textual features, algorithm and domain knowledge. Previous works using textual features to calculate similarity are mainly based on corpus-based methods such as bag-of-words and word embeddings. Bag-of-words model, including vector space model (VSM) [5], latent semantic analysis (LSA) [6], and latent Dirichlet allocation (LDA) [7], treats the entire text as a set of words and calculates the weight for each word, thus transforms them into real-valued vectors and then calculate the similarity on top of them [8–10]. These methods need handcrafted features and external lexical resources, which makes it difficult to apply in domains without too much readily available knowledge. Word embeddings are low-dimensional real-valued vectors trained from large-scale unlabeled text. Those vectors are able to capture semantic relationships among free text documents [4, 11].

Convolutional neural network (CNN) is a typical artificial neural network algorithm which could automatically learn, filter, cluster and combine features without much human effort. It was originally invented for computer vision [12] and subsequently been shown to be effective in natural language processing (NLP) tasks, such as sentence modeling [13], search query retrieval [14], and semantic parsing [15]. CNN models can nicely represent the hierarchical structures of sentences with their layer-by-layer convolutional kernel and pooling, so as to capture the semantic patterns at different layers [16]. CNN has also been demonstrated to be effective in capturing the semantic similarities between text pairs and thus able to perform well on text matching tasks [17, 18].

Moreover, domain ontologies contain lots of semantic relations which represent the body of knowledge. In the medical domain, there are a number of popular biomedical ontologies, such as MeSH (Medical Subject Headings) for indexing literature, the ICD taxonomy (International Classification of Diseases) for public health surveillance and ﻿billing purposes, and SNOMED-CT for aggregating medical terms across sites of healthcare. All these ontologies use graph structures [19] to represent the relationships among medical concepts. However, it is not straightforward to use this extra knowledge by conventional machine learning methods. Graph embedding technology embeds edge and node information of graphs into low dimensional dense vectors [20], and we believe it has a great potential to facilitate the utility of those ontologies.

In this study, we propose an end-to-end solution based on CNN to help physician and clinical quality control staff efficiently retrieve patients’ examination reports for imaging diagnosis verification process. The input of the model is imaging and pathology report-pairs from certain patients, and the output is the corresponding label indicating whether the report-pairs contain overlapping body sites. We compared accuracy of our model (with different word embedding methods) with conventional approaches such as keyword mapping, latent semantic analysis (LSA) [6], latent Dirichlet allocation (LDA) [7], Doc2Vec [21], Siamese LSTM [22] and a method based on named entity recognition (NER) [23]. Moreover, we further applied the LIME algorithm [24] to identify the features contribute most to the final results, and imporved the model interpretability.

## Methods

### Technical workflow

**Fig. 1:**
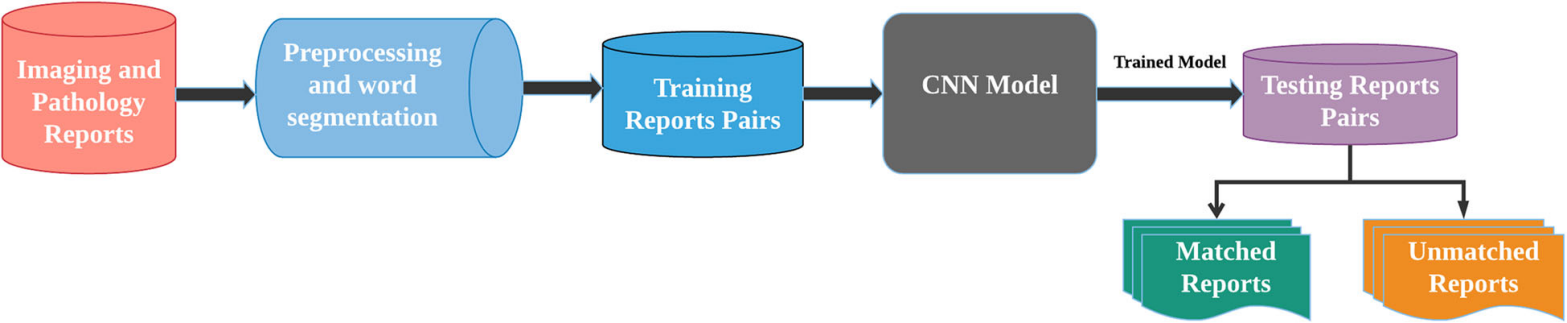
Workflow of detecting text semantic similarity

Figure 1 shows the workflow of identifying matching body sites from medical report-pairs. We removed all punctuations, numbers, and stop words from the raw report texts, then used Jieba,^1^ a Chinese segmentation tool, to transform entire texts into sequence of words for CNN model training. The study and data use were approved by the Human Research Ethics Committees of Tongren Hospital, Shanghai Jiao Tong University, Shanghai, China.

### Data description

We included 4262 imaging reports and 2141 pathology reports from the EMRs of 1926 patients who admitted to Shanghai Tongren Hospital and had ultrasonic examinations between 1st May 2017 and 31st July 2017, which finally resulted in 16,354 report-pairs. All report texts were de-identified. Each pair contained two pieces of report, one is imaging report and the other is pathology report. Three physicians were recruited to annotate whether each report-pair contains overlapping body sites independently, the kappa coefficient between each pair of two physicians is 0.95, 0.95, 0.97 respectively. The overall rate of positive pairs (which contain overlapping body sites) was 14.8% (2415/13,939). We randomly split the data into 80% for training and 20% for testing.

### CNN model for text similarity detection

**Fig. 2:**
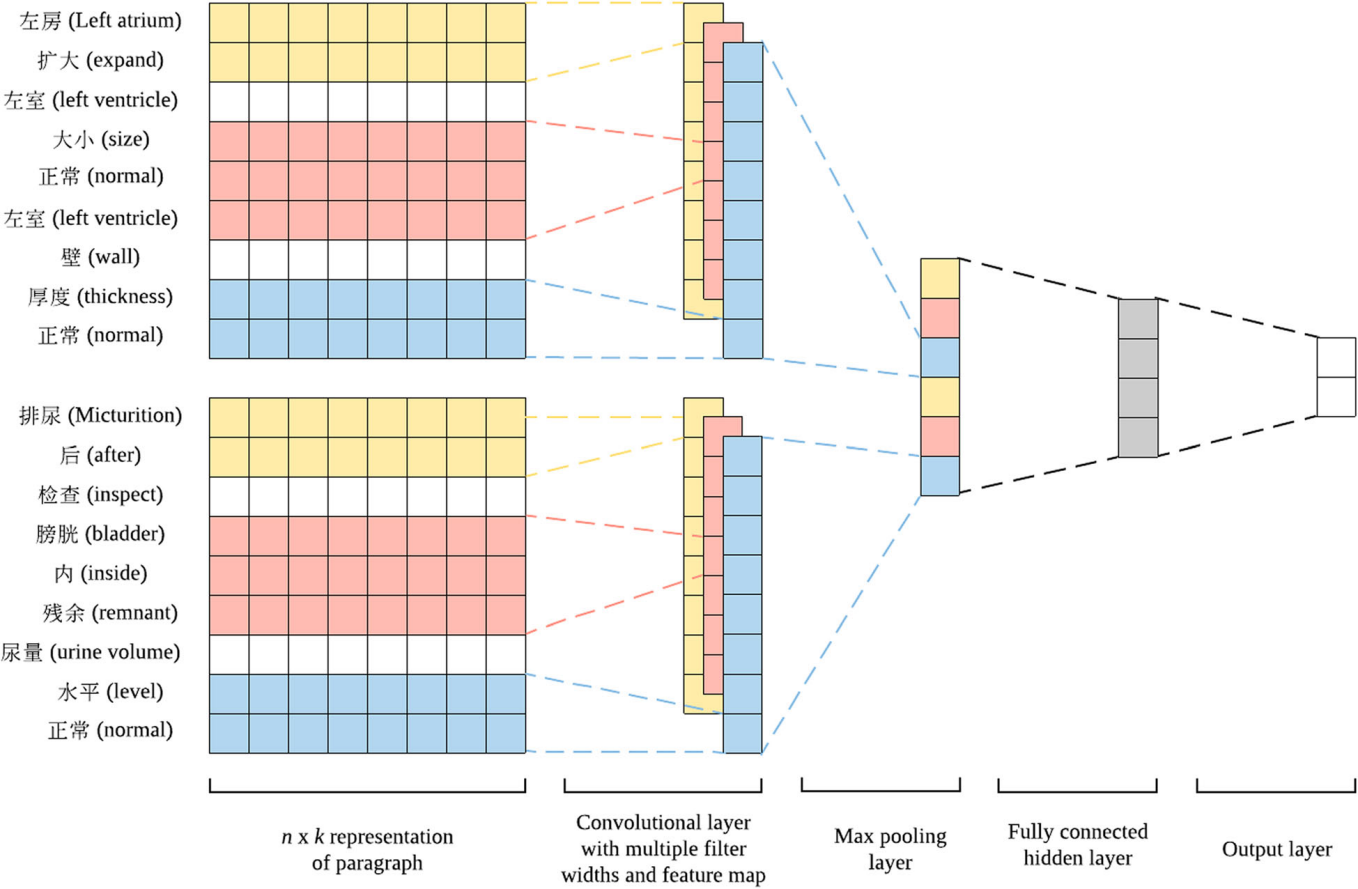
CNN-based neural network for text similarity detection

The structure of our model (showed in Fig. 2) can be divided into three parts: input layer, feature extraction layer and fully connected layer.

The input layer mapped each word into a dense vector (with 128 dimensions) and transformed each report into a dense matrix. Each dense vector represented the semantic information of corresponding word, the values of which could be updated during training. We used two strategies to initialize word vectors: randomly initialized vectors and pre-trained (word2vec model trained by skip-gram and negative sampling method) word vectors using Baidu Encyclopedia corpora obtained from github.^2^ We set the window length to be 3, 4 and 5, for the convolution filters, and adopted 32 convolution filters for each window size. Then we applied max-pooling operations and obtained a new feature vector for two reports respectively. We concatenated the two feature vectors and passed it to a fully connected layer and a output layer to calculate the likelihood of containing overlapping body sites. We set cross-entropy as the loss function and performed mini-batch stochastic gradient descent to train the model.

### Medical concept vectors using ontology-based graph embedding

We utilized graph embedding method as a third word vector initialization strategy to enhance the word representation by capturing the semantic relations information from medical ontologies. We used CMeSH (Chinese Medical Subject Headings), a Chinese version of MeSH, which contains about 391,892 medical concepts and 2,047,749 relations, to train our medical concept vectors. We randomly generated word sequences by sampling neighbor concepts along the edge of relation in CMeSH with a length of 10. The sampling process basically follows the procedure in node2vec [20], which was composed of two major steps: 1) for every node (medical concept) *V*, adding its direct (1st order) neighbors to the sampling set ${\mathcal{M}}_V$; 2) let *V*_*m*_ be the m-th order neighbor of *V* and ${V}_m^1$ be the direct neighborhood of *V*_*m*_, then we randomly sample one node from ${V}_m^1$ and add it to ${\mathcal{M}}_V$. In our experiments, we set m to be 9 and sampled a word sequence for each node. And we feed the sequence set ${\mathcal{M}}_V$ into word2vec model with skip-gram method [25] to train the medical concept vectors.

### Model evaluation

We compared the performance of our CNN model with the following six baseline models:

- Keyword mapping. We used the vocabulary from CMeSH as a medical dictionary to filter the original text. All words outside the dictionary were discarded and Jaccard similarity coefficient was calculated based on the key words remained in the two report texts
- Latent Semantic Analysis (LSA). For this approach we collected all reports and construct bag-of-words representation vectors for each of them. Then singular value decomposition was performed on the matrix concatenating all bag-of-words vectors to reduce the dimensionality of the vector representations and cosine similarity was measured on those vectors from the reduced-dimension space
- Latent Dirichlet Allocation (LDA). This approach constructed the bag-of-words representations for the reports. It assumed that each report was a mixture of a set of “topics” and each topic was a mixture of the set of words in the vocabulary. Cosine similarity was measured on their topic composition vectors.
- Doc2Vec. Doc2Vec is an extension to the Word2Vec model [26], where a document vector is trained together with the word vectors in the continuous bag-of-words model. Cosine similarity was measured on the learned document vectors.
- Siamese long short term memory (LSTM). Siamese LSTM is often used for text similarity systems. It uses two LSTM networks to encode two sentences respectively, then calculate Manhattan distance between the encoded hidden vectors to decide whether the two sentences are similar or not. The training process is supervised.
- Named Entity Recognition (NER). We used another annotated Chinese clinical EMR corpus from Shanghai Tongren Hospital. This corpus contains 46,665 sentences and 89,231 entities of four types: symptoms, diseases, lab tests and body structures. We trained a DNN-based NER model with random initialized word embedding [23] and then adopted this model to identify all the entities in the original report texts. We only keep these entity words and construct bag-of-words representation vectors for each of the reports. Cosine similarity was measured on their entity representation vectors.

All models were trained on the training set and evaluated on the testing set. We performed receiver operating characteristic (ROC) curve analysis for each model and calculated the AUC score. We calculated precision, recall and F1-score based on the cutoff value equal to the ratio of the positive pairs in the whole dataset. Report-pairs with similarity score higher than the cutoff value will be labeled positive in all of our models. We used bootstrapping method with 50 times repeated samplings to estimate mean and standard deviation (std) of our model performances. Because of data imbalance, we reported both overall performance (marco average) and performance for each class group.

### Model interpretability

We further explored the LIME algorithm to improve the interpretability of our model. LIME, proposed by Guestrin et al. [24], can be used to explain the predicted results of machine learning models. The basic idea of LIME algorithm is to define “interpretation” using another model, usually a linear model or a decision tree. We adopted LIME algorithm to identify which keywords from report-pairs our CNN model took to give final results. Specifically, for a given report-pair, we first fixed the content of imaging report and generated new samples of pathology report by randomly deleting words. Then, we trained a LIME model on the generated pairs and calculated the relative importance for each word in the pathology report. Similarly, we also fixed the content of the pathology report, randomly generated the pairs and trained another LIME model for the pathology report. We represented the relative importance of the keywords in a visual way.

## Results

### Model performance

**Fig. 3:**
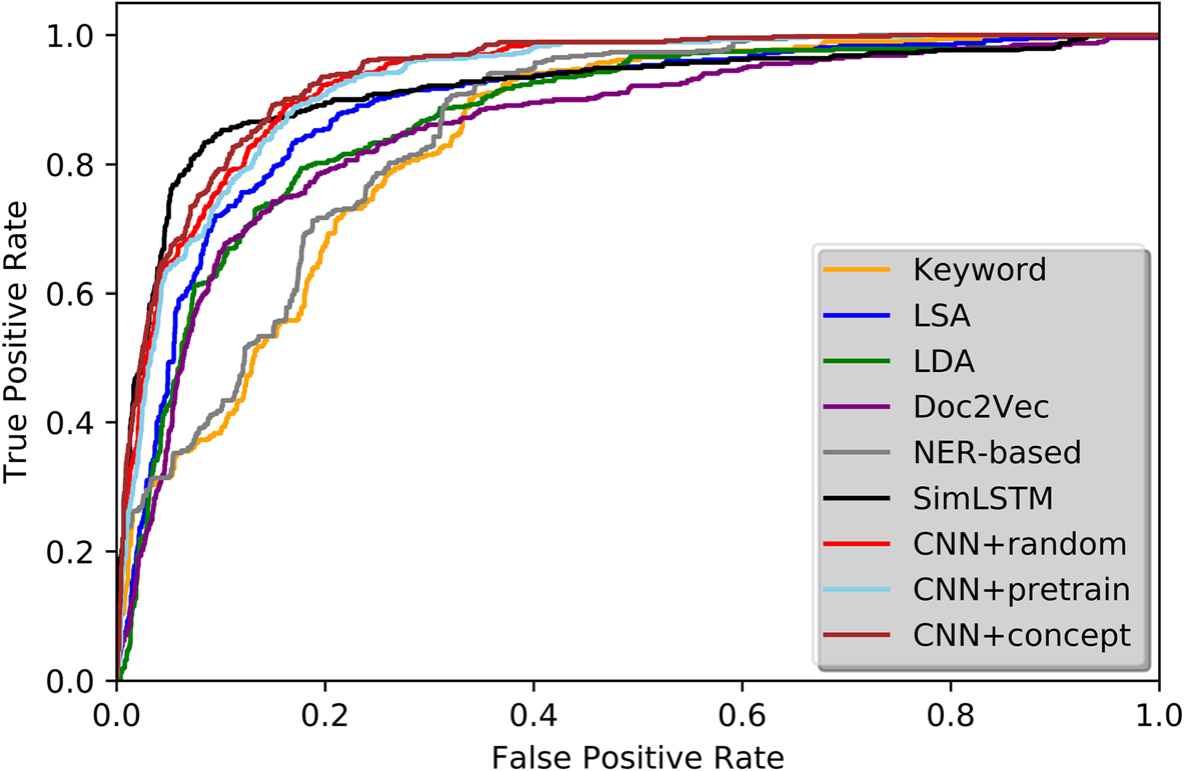
ROC Curve of different models

Table 2 showed both average and class-level performances for all models and Fig. 3 showed the corresponding ROC curves. The AUC score of our CNN models with both randomly initialized vectors and pre-trained word vectors were superior than that of any other baseline models, and gained approximately 3–7% improvement. In particular, the AUC score of CNN model with medical concept vectors was 0.8% higher than the model with randomly initialized vectors and 1.5% higher than the one with pre-trained word vectors. We have done t-test to the AUC results from 50 independent runs of CNN with or without pre-trained medical concept vectors and the *p*-value is smaller than 0.001, which suggests the improvement is significant. Not surprisingly, keyword mapping model had the worst performance among all models.

**Table 2:** Performance comparison of different models including Precision/Recall/F1-score

Model	Macro average			Positive class			Negative class			AUC (mean ± std)
	Precision (mean ± std)	Recall (mean ± std)	F1-score (mean ± std)	Precision (mean ± std)	Recall (mean ± std)	F1-score (mean ± std)	Precision (mean ± std)	Recall (mean ± std)	F1-score (mean ± std)	
Zero-r	0.73 ± 0.0	0.85 ± 0.0	0.78 ± 0.0	0.0 ± 0.0	0.0 ± 0.0	0.0 ± 0.0	0.85 ± 0.0	1.0 ± 0.0	0.92 ± 0.0	0.0 ± 0.0
Keyword Mapping	0.827 ± 0.006	0.842 ± 0.005	0.833 ± 0.005	0.464 ± 0.023	0.358 ± 0.018	0.404 ± 0.018	0.891 ± 0.004	0.927 ± 0.004	0.909 ± 0.003	0.840 ± 0.004
LSA	0.892 ± 0.005	0.862 ± 0.007	0.873 ± 0.006	0.512 ± 0.022	0.758 ± 0.019	0.611 ± 0.019	0.956 ± 0.004	0.879 ± 0.008	0.916 ± 0.005	0.894 ± 0.006
LDA	0.872 ± 0.006	0.852 ± 0.006	0.860 ± 0.006	0.514 ± 0.021	0.669 ± 0.023	0.581 ± 0.019	0.936 ± 0.005	0.884 ± 0.005	0.910 ± 0.004	0.879 ± 0.004
Doc2Vec	0.882 ± 0.007	0.862 ± 0.007	0.869 ± 0.007	0.514 ± 0.019	0.682 ± 0.023	0.586 ± 0.018	0.943 ± 0.005	0.892 ± 0.006	0.917 ± 0.004	0.871 ± 0.005
NER-based	0.835 ± 0.006	0.849 ± 0.005	0.842 ± 0.006	0.473 ± 0.022	0.501 ± 0.020	0.482 ± 0.020	0.904 ± 0.006	0.923 ± 0.005	0.912 ± 0.005	0.853 ± 0.004
Siamese LSTM	0.920 ± 0.006	0.891 ± 0.005	0.904 ± 0.006	0.582 ± 0.020	0.843 ± 0.021	0.698 ± 0.020	0.964 ± 0.006	0.901 ± 0.007	0.932 ± 0.006	0.916 ± 0.006
CNN + random vector	0.916 ± 0.005	0.931 ± 0.006	0.923 ± 0.005	0.631 ± 0.022	0.833 ± 0.019	0.712 ± 0.019	0.972 ± 0.007	0.917 ± 0.005	0.941 ± 0.005	0.942 ± 0.003
CNN + pretrain vector	0.912 ± 0.006	0.927 ± 0.006	0.920 ± 0.006	0.637 ± 0.021	0.811 ± 0.019	0.701 ± 0.020	0.965 ± 0.006	0.920 ± 0.005	0.937 ± 0.006	0.936 ± 0.004
CNN + concept vector	0.931 ± 0.006	0.938 ± 0.007	0.935 ± 0.006	0.682 ± 0.023	0.771 ± 0.020	0.734 ± 0.021	0.969 ± 0.004	0.938 ± 0.008	0.954 ± 0.007	0.951 ± 0.003

### LIME experiment

We randomly selected two report-pairs which contain overlapping body site in the test sets and process through LIME model. Table 3 shows the original text for two sample pairs (sample pair No. 1 and No. 2) and Table 4 shows the corresponding results. For sample pair No. 1, the importance scores of words, “fetal membrane” (胎膜) and “umbilical cord” (脐带) in the pathology report, and “fetus” (胎儿) and “fetal heart” (胎心) in the imaging report, were relatively high with a score of 0.15 and 0.14, 0.12 and 0.03 respectively. The result indicated that the existence of these four words might account for the positive judgement with a prediction of 0.77 by our CNN model. “Fetal membrane”, “umbilical cord” and “fetal heart” were all body structures contained by “fetus”, and our CNN model was able to automatically and reasonably extract semantic features from texts and make judgements. For sample pair No. 2, the word “Thyroid” (甲状腺) which exist in both reports, contributes most to the result, with a score of 0.16 and 0.19 respectively. “Tubercle” (结节) and “Glandular body” (腺体) are sub-structures of thyroid gland and also contributed much to the final result. The LINE algorithm could efficiently locate the most related words from text pairs and provide meaningful explanations of our model behaviors.

**Table 3:** The original text of selected samples

Sample pair No.	Imaging report content (Chinese)	Imaging report content (English)	Pathologic report content (Chinese)	Pathologic report content (English)
1	宫内见1个胎儿, 胎位头位, 胎方位LOP。双顶径81, 枕额径101, 腹前后径92, 腹左右径83, 股骨长60, 肱骨长52。胎心胎动见, 胎心133次/分, 胎心律齐。胎盘位于后壁, 厚度35, 分级II, 胎盘下缘距宫颈内口> 54。羊水指数31 + 31 + 36 + 39。胎儿脐血流指数:PI = 0.93, RI = 0.63, S/D = 2.71 单胎头位。胎儿迟发畸形的检查受多因素影响, 超声无法检出所有胎儿异常。此检查仅限于胎儿生长监测。	One fetus can be observed in the uterus. The position of the fetus is cephalic position, the orientation is LOP, the biparietal diameter is 81, the occipitofrontal diameter is 101, the anteroposterior trunk diameter is 92, the transverse trunk diameter is 83, the femur length is 60, the humeral length is 52. Fetal heart rate and fetal movement can be observed. The fetal heart rate us 133 beats per minute and the heart rhythm is regular. The placenta is located in the posterior wall. The thickness of the placenta is 35, grade II. The distance between the placental margin and the internal cervical os is > 54. The Amniotic fluid index is 31 + 31 + 36 + 39. Fetal umbilical artery plow index: PI = 0.93, RI = 0.63, S/D = 2.71. Singleton and cephalic presentation. The examination of fetal delayed malformation is affected by many factors, and ultrasound cannot detect all fetal abnormalities. This examination is limited to fetal growth monitoring.	胎盘组织重600g, 大小21*17*3 cm, 胎膜完整, 切面灰红色, 母面小叶完整, 子面光滑, 相连脐带长35cm, 直径1.2 cm, 血管三根。(胎盘)孕晚期胎盘一个, 绒毛发育良好, 脐带及胎膜未见明显异常。	The weight of placental tissue is 600 g, the size is 21 × 17 × 3 cm, the fetal mem-brane is intact, the cut sur-face is gray-red, the lobules of maternal surface are intact, and the daughter surface is smooth. The length of the umbilical cord is 35 cm, the diameter is 1.2 cm, and three blood vessels can be observed. (Placenta) favor a diagnosis of previa of late pregnancy, the villi are well-developed, and no obvious lesion is observed in umbilical cord and fetal membrane.
2	甲状腺大小正常, 包膜清晰完整, 内部回声分布均匀, CDFI:腺体内部血流信号未见明显异常。甲状腺右叶内可见数个低回声区, 大者大小23.5*13.2 mm, 形态规则, 边界清晰, 内部回声不均匀。	The size of thyroid gland is normal, the capsule is clear and intact, and the echogenicity is homogeneous. CDFI: There is no obvious abnormality of blood flow signal in the gland. There are several hypoechoic areas in the right lobe of the thyroid. The size of the lesion is 23.5 × 13.2 mm, the shape is regular, the boundary is clear, and the echogenicity is inhomogeneous.	甲状腺组织, 大小4.5*2.5*1.5 cm, 切面见结节两枚, 直径1-2 cm, 灰红色, 质软。(甲状腺右叶)结节性甲状腺肿伴滤泡性腺瘤形成。	The specimen for pathological examination contains one thyroid tissue. The size of the tissue is 4.5 × 2.5 × 1.5 cm. Two thyroid nodules can be observed from the cut surface. The diameter of the nodules is 1 to 2 cm, the color are grey red, the texture is soft. (The right lobe of the thyroid) favor a diagnosis of nodular goiter combined with follicular adenoma.

**Table 4:** Sample-level feature importance of sample pair 1 and 2 for both imaging and pathologic report provided by LIME algorithm

Sample pair No.	Imaging report			Pathologic report		
	Word-Chinese	Word-English	Feature importance of word	Word-Chinese	Word-English	Feature importance of word
1 (Prediction probability = 0.77)	胎膜	Fetal membranes	0.15	胎儿	Fetus	0.12
	脐带	Umbilical cord	0.14	胎心	Fetal heart	0.03
	胎盘	Placenta	0.06	羊水	Amniotic fluid	0.03
	毛发	Hair	0.04	头位	Head position	0.02
	小叶	Lobule	0.02	股骨	Femur	0.01
	面灰	Face ash	0.01	单胎	Single fetus	0.01
2 (Prediction probability = 0.83)	甲状腺	Thyroid	0.19	甲状腺	Thyroid	0.16
	结节	Tubercle	0.15	腺体	Glandular body	0.14
	滤泡	Follicular	0.08	右叶	Right lobe	0.07
	右叶	Right lobe	0.03	包膜	Envelope	0.03
	腺瘤	Adenoma	0.01	回声	Echoes	0.01
	切面	Section	0.01	血流	Blood flow	0.01

## Discussion

In this paper, we proposed a direct end-to-end CNN model to judge whether two reports contained matching body sites. Comparing with conventional language models based on handcrafted textual features (keywords and bag-of-words), automatically generated features (bag-of-words extracted by our NER model and word embeddings) and neural network model with LSTM structure, our CNN model provided more flexibility in exploring the semantic information contained in medical documents and yield better performance. In addition, we compared three strategies to generate word vectors for our CNN model: randomly initialized vectors, pre-trained word vectors and graph embedding and CMeSH based medical concept vectors. Our CNN model with medical concept vectors outperformed the other two methods and an significant improvement was observed.

Many factors might contribute to the advantage of CNN model. First, our CNN model is a supervised learning model and could automatically adapt feature representations to task objectives. For LSA, LDA and Doc2Vec, we learn feature representations in an unsupervised way, semantic information and co-occurrence relationship of words or characters are weakly correlated with current learning objectives. Second, our CNN model could extract syntactic and semantic information from both local semantic patterns and hierarchical structures of the sentences. For example, body sites could be described by physicians using anatomy terms and their relative locations. Thus, information at word-level or chunk-level is more important than information at sentence-level or document-level. This could explain why the performance of our CNN model was higher than Siamese LSTM model. Even though Siamese LSTM performs better on precision than CNN model with randomly initialized vectors, it’s F1-score was significantly lower than our CNN. Third, we used end-to-end training strategy, which updated feature representations and optimized weights simultaneously.

We used graph embedding method to utilize domain knowledge from CMeSH and gained a significant performance boost. CMeSH, just like other domain-specific ontologies, organizes and represents the body of knowledge using concepts and their relations. For example, concept “parotid gland” is a sub-class of concept “salivary glands”, concept “salivary glands” is a sub-class of both concepts “exocrine glands” and “mouth”, and concept “salivary glands” contains sub-class concepts “parotid gland”, “salivary ducts” and “sublingual gland”. In our study, the affiliation information of anatomy terms extracted by graph embedding method was quite useful to judge overlapping body sites, and could explain for the higher performance.

To validate that our model could correctly find related semantic or anatomy information and make judgement as expected, we used LIME algorithm and analyzed two concrete examples. From the results we could see our CNN model chose reasonable keywords as the basis to give the predictions. In real world, we can incorporate these explanations of model behaviors into the computer-aided decision supporting system so as to further remind the clinical quality control staff why our model give such results.

Our CNN model still has several limitations. We performed an error analysis of our model and find several typical mis-classifications. Table 5 shows two sample pairs from the analysis, sample pair No.3 is a case of false-negative and No.4 is a case of false-positive. Sample pair No.3 indicates that our model could not correctly identify spatial relationship between body structures. In sample pair No.3, the imaging report described a mass observed in the ventral side of the inferior pole of left kidney, and the pathologic report described a lesion from left adrenal gland. This report-pair does not share common anatomical terms, but both left kidney and adrenal gland are adjacent body structures in local anatomy space, and thus the report-pair shares common body site. Sample pair No.4 indicate that our model is insensitive to direction information. In sample pair No.4, both imaging report and pathology report described the lymph node of armpit, but the one of imaging report is from left armpit and the one of pathology report is from right armpit, and thus this report-pair share no common region. There are other limitations including: first, Chinese word segmentation using Jieba was imperfect and might induce errors in segmentations, especially for medical terms; second, there is no Chinese version of SNOMED-CT or UMLS (Unified Modeling Language), and thus we only performed graph embedding on CMeSH, which has relatively small number of concepts and relations; third, we only evaluated our model on Chinese medical reports, but it could be easily move onto other language scenarios without language-specific optimizations.

**Table 5:** Sample pairs from error analysis

Sample pair No.	Imaging report content (Chinese)	Imaging report content (English)	Pathologic report content (Chinese)	Pathologic report content (English)	True label	Predict label
3	于左肾下极腹侧可见多个囊性为主的混合性回声, 相互融合, 较大之一约17.1 × 17.0 mm(局部凸向肾外), 靠近肾盏之一大小约14.2 × 14.6 mm, 形态欠规则, 表面光整, 境界欠清, 囊内无回声透声尚可, 分布欠均, 可见分隔样回声, 间隔及囊壁未见明显增粗, 囊内及囊壁可见点状、带状强回声, 团块后方回声无明显改变, CFI示未见明显血流信号。	Multiple cystic mixed echoes can be observed in the ventral side of the inferior pole of left kidney, which fuse with each other. The largest one is about 17.1 × 17.0 mm (which protrudes out locally from the kidney), and the one near the renal pelvis is about 14.2 × 14.6 mm. The shape of the cysts is irregular, the surface is smooth, and the boundary is not clear. There are no echoes in the cysts, the sound transmission is normal, but the echogenicity is inhomogeneous, and septations can be observed. There is no obvious thickening for both septations and walls of the cysts. Punctate and banded strong echoes can be observed inside the cysts and on the wall of the cysts. There is no obvious lesion behind the cysts, and CFI showed no obvious blood flow signal.	肿物两枚, 直径1cm, 暗黄色, 质中。另见肾上腺组织, 大小2.5*1.5*1.5 cm, 暗红色, 质中。(左肾上腺)倾向皮质结节状增生。	The specimen for pathological examination contains two masses and one adrenal tissue. The diameter of masses is 1 cm, the color is dark yellow, and the texture is medium level. The size of adrenal tissue is 2.5 × 1.5 × 1.5 cm, the color is dark red, and the texture are medium level. (Left adrenal gland) favor a diagnosis of nodular adrenal cortical hyperplasia.	True	False
4	左侧腋下见数个淋巴结样回声区, 大者11mm*5 mm, 边界清, 有包膜, 形态规则, 内部结构清晰, 未见明显血流信号。左侧腋下可见多个淋巴结。	There are several lymphoid echoes under the left armpit, the largest one is 11 mm × 5 mm, the boundary is clear, the capsule is regular, the internal structure is clear. There is no obvious blood flow signal. Multiple lymph nodes can be seen in the left armpit.	脂肪组织, 大小3.5*3*1 cm, 找见淋巴结两枚。(右腋下淋巴结)淋巴结(0/1)未见癌转移。免疫组化:(右腋下淋巴结)淋巴结(0/1)未见癌转移。	The specimen for pathological examination contains one fat tissue. The size of fat tissue is 3.5 × 3 × 1 cm, and two lymph nodes can be seen in the tissue. (The lymph node of right armpit) lymph node (0/1) show no metastasis. Immunohistochemical staining method: (the lymph node of right armpit) lymph node (0/1) show no metastasis.	False	True

In this paper we only focused on identifying whether a pair of reports contain overlapping body sites. We treated it as a binary classification problem, trained a CNN model and used graph embedding based on CmeSH ontology. In future, we could: first, consider it as a ranking problem, annotate and train a machine learning model to identify whether report A is more similar to report B than report C (e.g., by checking the number of overlapping body parts); second, try different graph embedding methods and combination of medical ontologies; third, validate the end-to-end architecture in other language tasks.

The proposed technique in this paper can be used for matching the reports of medical images from different resources and help better consolidate the heterogeneous patient clinical information and improve the efficiency of clinicians. Fundamentally, our study provides a generalizable architecture to detect information discrepancies from different sources of routinely collected clinical data. With the increasing secondary use of clinical data, many commercial software was developed for similar purpose but with different data sources and algorithms, for example, IBM Watson Imaging Clinical Review.^3^ Moreover, as Wang et al. [27] have envisioned, improving the quality of clinical data is one key aspect to make artificial intelligence tools really useful in clinical practice. The effective consolidation of clinical data can help us better reconcile them, detect potential errors and thus improve the data quality.

## Conclusion

In this paper we proposed a convolutional neural network-based model to identify report-pairs of imaging examinations and pathologic examinations which contain overlapping exam body sites by detecting semantic similarity. Our model exhibited superior performance compared to other conventional models such as key word mapping, LSA, LDA, Doc2Vec, Siamese LSTM and a method based on NER. We also leveraged graph embedding method to utilize external information from medical ontologies and gained further improvement. In addition, we adopted LIME algorithm to analyze our model behavior in a visible way. The results indicated our model was able to automatically and reasonably extract semantic features from texts and make accurate judgements. It could help retrieve patients or reports for imaging diagnosis quality measurement in a more efficient way.

## Data Availability

Although we obtained permission from the institutional ethics committee to use the data and enforced a degree of data de-privacy, we did not obtain informed consent from patients to disclose medical history data. Therefore, in China where the HIPAA Act does not exist, we cannot determine what extent the de-privatization of data sharing conforms to Chinese laws and regulations without the consent of patients. In view of legal risks, we hope not to share data publicly. But we will do our best to help other scholars who are interested in our research and hope to reproduce the results.

## Abbreviations

AUC: Area under the ROC curve
CMeSH: Chinese Medical Subject Headings
CNN: Convolutional neural network
Doc2Vec: Document to vector method
EMRs: Electrical medical records
ICD: International Classification of Diseases
LDA: Latent Dirichlet allocation
LSA: Latent semantic analysis
LSTM: Long short term memory
MeSH: Medical Subject Headings
NER: Named Entity Recognition
NLP: Natural language processing
VSMs: Vector space models

## Footnotes

1
https://github.com/fxsjy/jieba

2
https://github.com/Embedding/Chinese-Word-Vectors

3
https://www.ibm.Com/blogs/watson-health/introducing-ibm-watson-imaging-clinical-review
